# Comparative Evolutionary Histories of Kisspeptins and Kisspeptin Receptors in Vertebrates Reveal Both Parallel and Divergent Features

**DOI:** 10.3389/fendo.2012.00173

**Published:** 2012-12-26

**Authors:** Jérémy Pasquier, Anne-Gaëlle Lafont, Hervé Tostivint, Hubert Vaudry, Karine Rousseau, Sylvie Dufour

**Affiliations:** ^1^Research Unit BOREA, Biology of Aquatic Organisms and Ecosystems, Centre National de la Recherche Scientifique 7208, Institut de Recherche pour le Développement 207, Université Pierre et Marie Curie, Muséum National d’Histoire NaturelleParis, France; ^2^UMR 7221 CNRS/MNHN Evolution des Régulations Endocriniennes, Muséum National d’Histoire NaturelleParis, France; ^3^Laboratory of Cellular and Molecular Neuroendocrinology, INSERM U982, European Institute for Peptide Research (IFRMP 23), University of RouenMont-Saint-Aignan, France

**Keywords:** kisspeptin, kisspeptin receptor, phylogeny, synteny, evolutionary history, spotted gar, coelacanth, European eel

## Abstract

During the past decade, the kisspeptin system has been identified in various vertebrates, leading to the discovery of multiple genes encoding both peptides (Kiss) and receptors (Kissr). The investigation of recently published genomes from species of phylogenetic interest, such as a chondrichthyan, the elephant shark, an early sarcopterygian, the coelacanth, a non-teleost actinopterygian, the spotted gar, and an early teleost, the European eel, allowed us to get new insights into the molecular diversity and evolution of both *Kiss* and *Kissr* families. We identified four *Kissr* in the spotted gar and coelacanth genomes, providing the first evidence of four *Kissr* genes in vertebrates. We also found three *Kiss* in the coelacanth and elephant shark genomes revealing two new species, in addition to *Xenopus*, presenting three *Kiss* genes. Considering the increasing diversity of kisspeptin system, phylogenetic, and synteny analyses enabled us to clarify both *Kiss* and *Kissr* classifications. We also could trace back the evolution of both gene families from the early steps of vertebrate history. Four *Kissr* and four *Kiss* paralogs may have arisen *via* the two whole genome duplication rounds (1R and 2R) in early vertebrates. This would have been followed by multiple independent *Kiss* and *Kissr* gene losses in the sarcopterygian and actinopterygian lineages. In particular, no impact of the teleost-specific 3R could be recorded on the numbers of teleost *Kissr* or *Kiss* paralogs. The origin of their diversity *via* 1R and 2R, as well as the subsequent occurrence of multiple gene losses, represent common features of the evolutionary histories of *Kiss* and *Kissr* families in vertebrates. In contrast, comparisons also revealed un-matching numbers of *Kiss* and *Kissr* genes in some species, as well as a large variability of *Kiss/Kissr* couples according to species. These discrepancies support independent features of the *Kiss* and *Kissr* evolutionary histories across vertebrate radiation.

## Introduction

As increasing vertebrate genomes have been explored, the understanding of their structure and evolution has progressed in parallel. Indeed, the comparison of their gene organization shed light on various large-scale genomic events that occurred along vertebrate radiation. Among those events, in the early stages of their history, vertebrates experienced two rounds of whole genome duplication (1R and 2R), resulting in fourfold-replicated genomes (Dehal and Boore, 2005; Van de Peer et al., 2010). These two events can be traced through the study of gene families currently presenting up to four paralogs. In addition, the comparison of teleost genomes with other vertebrate genomes revealed a teleost-specific third round of whole genome duplication (3R), resulting in up to eight paralogs in the same gene family in this lineage (Amores et al., 1998; Meyer and Van de Peer, 2005; Kasahara et al., 2007).

In 1996, kisspeptin was first discovered as an anti-metastatic peptide in human carcinoma (Lee et al., 1996). In 2001, the orphan receptor GPR54 was identified as the cognate receptor of kisspeptin (Kotani et al., 2001; Muir et al., 2001). Two years later, both kisspeptin (Kiss) and its receptor (Kissr) were demonstrated as key players of the reproductive function in mammals (de Roux et al., 2003; Funes et al., 2003; Seminara et al., 2003). They act up-stream in the gonadotropic axis mediating gonadotropin releasing hormone (GnRH) and steroid effects on gonadotropin secretion, and are considered as major puberty gatekeepers and reproduction regulators (Pinilla et al., 2012). To date, the kisspeptin system has been identified in various vertebrate species, leading to the discovery of multiple genes encoding Kiss as well as multiple genes encoding Kissr (Biran et al., 2008; Felip et al., 2009; Kitahashi et al., 2009; Lee et al., 2009).

Concerning *Kiss* and *Kissr* diversity, contrasting situations are found in the different vertebrate phyla. Indeed, in eutherian species, one single gene, named *Kiss1r*, encodes the kisspeptin receptor and one single gene, named *Kiss1*, encodes kisspeptin. In prototherians, such as platypus (*Ornithorhynchus anatinus*), two *Kiss*, and two *Kissr* are present (Lee et al., 2009). To date, in teleosts, two situations have been reported. One *Kiss* gene and one *Kissr* gene are present in some species such as fugu (*Takifugu niphobles*), tetraodon (*Tetraodon nigroviridis*), and stickleback (*Gasterosteus aculeatus*). In contrast, a second *Kiss* as well as a second *Kissr* genes have been characterized in some species including zebrafish (*Danio rerio*; Biran et al., 2008), goldfish (*Carassius auratus*; Li et al., 2009), medaka (*Oryzias latipes*; Lee et al., 2009), and striped bass (*Morone saxatilis*; Zmora et al., 2012). Until recently the maximum number of *Kiss* and *Kissr* genes was found in an amphibian species, the *Xenopus* (*Xenopus tropicalis*), with three paralogs of each gene. On the opposite, in birds (chicken, *Gallus gallus*, zebra finch, *Taeniopygia guttata*, and turkey, *Meleagris gallopavo*) neither *Kiss* nor *Kissr* have been found. So far, in all these cases, a matching number of *Kiss* and *Kissr* genes had been reported, leading to the suggestion of the occurrence of “paired Kiss/Kissr” systems in vertebrates (Kim et al., 2012).

The recent publications of genomes from representative species of key phylogenetic positions makes it possible to revisit the diversity, the origin and the evolution of *Kiss* and *Kissr* in vertebrates. These genomes include a chondrichthyan, the elephant shark (*Callorhinchus milii*; Venkatesh et al., 2007), a representative of early sarcopterygian, the coelacanth (*Latimeria chalumnae*, coelacanth genome project, Broad Institute), a non-teleost actinopterygian, the spotted gar (*Lepisosteus oculatus*; Amores et al., 2011), and an early teleost (elopomorphe), the European eel (*Anguilla anguilla*; Henkel et al., 2012). Gene characterization, phylogenetic, and syntenic analyses allowed us to provide new insights on the respective evolutionary histories of *Kiss* and *Kissr* families. Furthermore, the comparison of proposed *Kiss* and *Kissr* phylogenetic histories highlighted common processes as well as divergent events leading to discuss the existence of conserved Kiss/Kissr couples among the various vertebrate lineages.

## Materials and Methods

### Genomic databases

The following genomic databases were investigated:

– the chicken genome^1^,
– the coelacanth genome^2^,
– the elephant shark genome^3^,
– the European eel genome^4^,
– the human genome^5^,
– the lizard genome^6^,
– the platypus genome^7^,
– the sea lamprey genome^8^,
– the spotted gar genome^9^,
– the stickleback genome^10^,
– the *Xenopus* genome^11^,
– the zebrafish genome^12^.

### TBLASTN search

The TBLASTN algorithm of the CLC DNA Workbench software (CLC bio, Aarhus, Denmark) was used on the European eel genome database and the elephant shark genome database. The TBLASTN algorithm (search sensitivity: near exact matches) of the *e*!ENSEMBL website^13^ was used on the coelacanth and spotted gar genomic databases.

### Gene predictions

**Figure 1:**
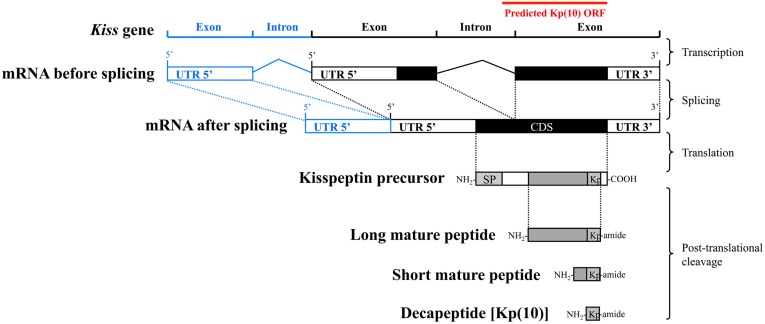
**Main steps leading from the *Kiss* gene to the Kp(10) peptide**. ORF, open reading frame; UTR, un-translated region; CDS, coding sequence; SP, signal peptide; Kp, mature kisspeptin. Predicted ORF sequence containing Kp(10) is represented as a red line. The intron and exon represented in blue have been observed in some mammalian *Kiss1* genes including human *Kiss1*, pig *Kiss1*, and mouse *Kiss1*.

Considering that *Kissr* gene structure as well as coding sequences (CDS) are well conserved among vertebrate species, it was possible to predict the exon and intron sequences for new *Kissr* genes (Pasquier et al., 2012). The splicing junctions were predicted using the empirical nucleotidic splicing signatures, i.e., intron begins with “GT” and ends with “AG.” Concerning *Kiss* structures, the fact that their CDS are split on two exons (Figure 1), appear to be conserved across vertebrates (for review: Tena-Sempere et al., 2012). However, they are highly variable among species, except for the sequence encoding the Kp(10) localized on the final exon (Figure 1). Therefore, only this Kp(10) conserved sequence can be predicted when investigating new genomes. This small sequence was used to identify the open reading frame (ORF) encompassing a part of the putative *Kiss* final exon and a part of the putative intron sequence (Figure 1). We therefore focused on this ORF encompassing the sequence encoding Kp(10), in the various genomes. The ORFs of the European eel, the coelacanth, the spotted gar, and the elephant shark, were determined using ORF finder tool of the CLC DNA Workbench software.

### Syntenic analyses

The synteny analyses of the eel genomic regions were manually performed using CLC DNA Workbench 6 software and the European eel genome database. The analyses of the spotted gar genomic regions were performed using the preliminary gene annotation of the genome assembly LepOcu1 generated by Ensembl release 67. Synteny maps of the conserved genomic regions in human, platypus, lizard (*Anolis carolinensis*), *Xenopus*, zebrafish, medaka, stickleback, tetraodon, and coelacanth, as well as of the corresponding region in chicken, *G. gallus*, were performed using the PhyloView of Genomicus v67.01 web site^14^ (Muffato et al., 2010).

## Results and Discussion

As one of the aims of this study was to compare the *Kiss* and *Kissr* histories, we first propose to make a short overview of our recent findings concerning the diversity, classification, and origin of *Kissr* gene family. Then, we will expose and discuss our new findings concerning *Kiss* family. Finally, we will compare and discuss the *Kiss* and *Kissr* evolutionary histories in order to get a better understanding of the kisspeptin system evolution.

### Diversity and evolutionary history of *Kissr* in vertebrates

#### Diversity and classification of *Kissr*

##### New advances in *Kissr* gene characterization

Recently, we described three *Kissr* genes in the genome of a basal teleost, the European eel, providing the first evidence of the existence of three *Kissr* genes in a teleost species (Pasquier et al., 2012). Furthermore, we described four *Kissr* in the genome of a non-teleost actinopterygian, the spotted gar, as well as in the genome of a basal sarcopterygian, the coelacanth (Pasquier et al., 2012). This provided the first evidence for four *Kissr* genes in vertebrate species and revealed a larger diversity of *Kissr* than previously described.

So far, no *Kissr* sequence has been reported in chondrichthyans. Our search in the elephant shark genome has only led to the identification of multiple partial sequences, corresponding at least to two *Kissr* (unpublished data). Ongoing sequencing of other chondrichthyan genomes, such as dogfish (*Scyliorhinus canicula*), may provide more insights into the *Kissr* diversity in the sister group of osteichthyans.

#### Phylogeny, synteny, and classification of *Kissr*

Phylogenetic analysis of 51 peptidic Kissr sequences clustered the osteichthyan Kissr into four clades, each one encompassing a coelacanth and spotted gar Kissr (Pasquier et al., 2012). Clade-1 mainly encompassed mammalian Kissr including human Kiss1r, as well as *Xenopus* Kissr-1a, European eel Kissr-1, spotted gar Kissr-1, and coelacanth Kissr-1. Clade-2 mainly encompassed teleost Kissr including European eel Kissr-2, as well as amphibian, spotted gar, and coelacanth Kissr-2. Clade-3 encompassed a few teleost Kissr including European eel Kissr-3 as well as *Xenopus* Kissr1b, spotted gar, and coelacanth Kissr-3. Clade-4 encompassed two early osteichthyan (spotted gar and coelacanth) Kissr-4 and two tetrapod Kissr (lizard and platypus; Pasquier et al., 2012).

Synteny analysis of *Kissr* neighboring genes, performed on 11 representative vertebrate species including the European eel, coelacanth, and spotted gar, fully supported the phylogenetic repartition of Kissr in four clades. Based on this classification, we proposed a new nomenclature of the *Kissr* family (*Kissr-1*, *Kissr-2*, *Kissr-3*, and *Kissr-4*; Pasquier et al., 2012).

### Evolutionary history of *Kissr*

#### Origin of *Kissr* diversity via 1R and 2R

**Figure 2:**
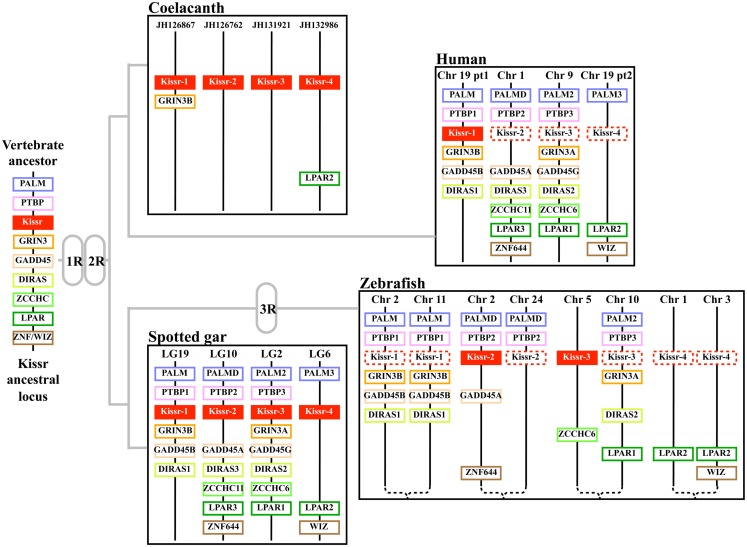
**Proposed origin of osteichthyan *Kissr* tetra-paralogons**. The paralogous genes of each of the eight identified families delineate four paralogons in the spotted gar, coelacanth, and human genomes and a duplicated tetra-paralogon in the zebrafish genome. This suggests a common origin of the four *Kissr* before the two whole genome duplication rounds (1R and 2R) which occurred in the early vertebrate history. This also suggests no impact of the teleost-specific 3R on *Kissr* number in current teleosts. Chr, chromosome; LG, linkage group.

Synteny analysis revealed that the four *Kissr* neighboring genomic regions were highly conserved, each presenting paralogs from eight gene families, i.e., *PALM*, *PTBP*, *GRIN3*, *GADD45*, *DIRAS*, *ZCCHC*, *LPAR*, *ZNF644/WIZ* (Pasquier et al., 2012). The hypothesis of the potential existence of four *Kissr* paralogons in vertebrates had been previously raised by Lee et al. (2009) and Kim et al. (2012), although only a maximum of three *Kissr* genes had been discovered at that time. Our finding of four *Kissr* genes, located on four paralogous genomic regions, in coelacanth and spotted gar, provides direct evidence validating this former hypothesis. These four *Kissr* paralogons likely resulted from the two successive genomic duplications (1R and 2R) of a single ancestral genomic region (Figure 2).

The currently available data led to a polytomy of the four Kissr clades. This polytomy did not allow to fully solve the homology relationships between the four *Kissr* resulting from the 2R (Pasquier et al., 2012). A recent study proposed the phylogenetic reconstruction of the *PALM* family (Hultqvist et al., 2012). The study of these genes, located in the vicinity of *Kissr* genes, allows us to infer further relationships between the four *Kissr*. We can thus hypothesize that *Kissr-1* and *Kissr-3*, on one side, and *Kissr-2* and *Kissr-4*, on the other side, could be sister genes resulting from the 2R.

Recently, one study proposed the reconstruction of 10 proto-chromosomes of the ancestral vertebrate karyotype and their linkage to the corresponding tetra-paralogons in the human genome (Nakatani et al., 2007). Considering our localization of the four *Kissr* syntenic regions in the human genome, we can hypothesize that the corresponding tetra-paralogons resulted from the duplications of one single region localized on the proto-chromosome-A of the vertebrate ancestor (Pasquier et al., 2012).

#### A subsequent history of *Kissr* losses

**Figure 3:**
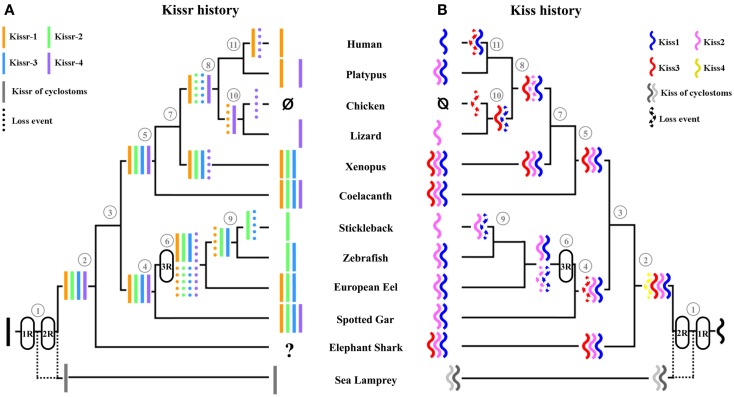
**Current status and proposed evolutionary history of *Kissr* genes (A) versus *Kiss* genes (B)**. (1) Vertebrates, (2) gnathostomes, (3) osteichthyans, (4) actinopterygians, (5) sarcopterygians, (6) teleosts, (7) tetrapods, (8) amniotes, (9) euteleosts, (10) diapsides, (11) mammals. The names of the current representative species of each phylum are given at the end of the final branches, together with the symbol of the *Kiss* and *Kissr* genes they possess. These hypotheses assume the presence of four *Kiss* and four *Kissr* paralogs in the vertebrate lineage resulting from the two rounds of vertebrate whole genome duplication. Multiple subsequent gene loss events are indicated in the various lineages.

Since the spotted gar and the coelacanth are the only two vertebrate species in which we discovered four paralogous *Kissr*, we can hypothesize multiple *Kissr* loss events to explain the status of this receptor in current vertebrates (Figure 3A). In the sarcopterygian lineage, *Kissr-4* may have been lost in amphibians, while *Kissr-1* and *Kissr-2* would have been lost in early amniotes. Subsequent additional losses may have led to the presence of only *Kissr-1* in eutherian mammals and to the absence of any *Kissr* in birds.

Considering the presence of four *Kissr* in a non-teleost actinopterygian, the spotted gar, the teleost-specific 3R could have resulted in the potential existence of up to eight *Kissr* genes. However, we only found three *Kissr* in the European eel, representing the current maximum number of this gene in teleosts. Furthermore, each eel *Kissr* is orthologous to a different tetrapod *Kissr*, supporting the absence of any teleost-specific *Kissr*. Synteny analysis demonstrated that each of the four *Kissr* paralogous genomic regions, present in the spotted gar, was duplicated in zebrafish, in agreement with the 3R. This analysis also indicated that all 3R-copies of *Kissr* were lacking in the corresponding duplicated regions (Figure 2). This suggests an early loss of duplicated *Kissr* genes, which would have suppressed the impact of the 3R on the number of *Kissr* in teleosts (Figure 3A). Additional successive deletions may have led to the presence of three *Kissr* in a basal teleost (the eel), two *Kissr* in a cypriniform (zebrafish), and only one Kissr in a more recent teleost (stickleback; Figure 3A).

### Diversity and evolutionary history of *Kiss* in vertebrates

In contrast to the receptor proteins which present several conserved domains, the *Kiss* genes encode precursors which are highly variable among vertebrates, except for the short sequence of the mature decapeptide [Kp(10)]. This makes it difficult to obtain *Kiss* mRNA sequences by classical molecular strategies. Genomic database analyses thus represent the best approach to characterize the *Kiss* set for a given species. However, the small characteristic sequence of *Kiss*, encoding Kp(10), could be missing in genomic databases due to sequencing or assembly limitations.

All previously investigated osteichthyan species possessed the same number of *Kiss* and *Kissr* genes: one in eutherian mammals, two in prototherians, three in *Xenopus*, none in birds, and one or two in teleosts (Lee et al., 2009). In cyclostomes, two *Kiss* genes have been reported (Lee et al., 2009), while only one *Kissr* could be predicted until now (Pasquier et al., 2011).

#### Diversity and classification of *Kiss*

##### New advances in *Kiss* gene characterization

**Figure 4:**
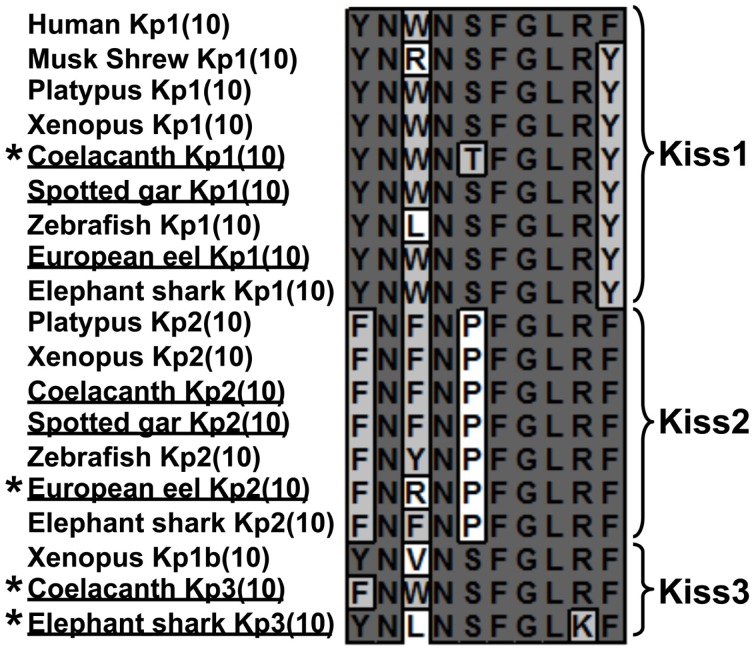
**Sequence alignment of vertebrate Kp(10)**. At each position, identical amino-acids are shaded in dark gray and similar amino-acids in light gray. Newly identified sequences are underlined and unique sequences are marked by an asterisk.

To further assess the *Kiss* diversity in vertebrates, we re-investigated the presence of these genes in the genome of the elephant shark, the coelacanth, the spotted gar, and the European eel, representative species from four groups of relevant phylogenetical positions. Most of the vertebrate *Kiss* genes are made of two exons except for some mammalian *Kiss1*, including human *Kiss1*, pig (*Sus scrofa*) *Kiss1*, and mouse *Kiss1*, that are made of three exons (Figure 1). However, the CDS of all the *Kiss* described so far are split on two exons (Figure 1). In fact the first of those two exons encodes the signal peptide while the final exon encodes mainly the mature peptides including the conserved Kp(10) (Figures 1 and 4; Tomikawa et al., 2010, 2012; Cartwright and Williams, 2012; Tena-Sempere et al., 2012). Considering that the *Kiss* gene sequences are highly variable among species except for the sequence encoding the Kp(10), we focused our prediction on the ORF containing this sequence. We performed TBLASTN in the four genomes, resulting in the identification of several ORF containing conserved sequences encoding for Kp(10).

*Two *Kiss* genes in the European eel genome*. The two ORFs containing the sequences encoding Kp(10) are 296 and 327 bp long, respectively (Figure A1 in Appendix). Once translated, each of them leads to a peptidic sequence encompassing a putative Kp(10): YNWNSFGLRY [European eel Kp1(10)] and FNRNPFGLRF [European eel Kp2(10)], respectively (Figure 4). The C-terminal end of the Kp1(10) sequence is followed by a GK-Stop motif, while the Kp2(10) sequence is followed by a GKR motif (Figure A1 in Appendix). The sequences “X-G-Basic-Basic” or “X-G-Basic” are characteristic of the conserved proteolytic cleavage and alpha-amidation sites of neuropeptides (Eipper et al., 1992).

*Two *Kiss* genes in the spotted gar genome*. The two ORFs containing the sequences encoding Kp(10) are 348 and 300 bp long, respectively (Figure A2 in Appendix). Once translated, each of them leads to a peptidic sequence presenting a putative Kp(10): YNWNSFGLRY [spotted gar Kp1(10)] and FNFNPFGLRF [spotted gar Kp2(10)], respectively (Figure 4). The C-terminal ends of these two sequences are followed by a GKR motif (Figure A2 in Appendix).

*Three *Kiss* genes in the coelacanth genome*. The three ORFs containing the sequences encoding Kp(10) are 363, 396, and 81 bp long, respectively (Figure A3 in Appendix). Once translated, each of them leads to a peptidic sequence encompassing a putative Kp(10): YNWNTFGLRY [coelacanth Kp1(10)], FNFNPFGLRF [coelacanth Kp2(10)], and FNWNSFGLRF [coelacanth Kp3(10)], respectively (Figure 4). The C-terminal ends of the Kp1(10) and the Kp2(10) sequences are followed by a GKR motif, while the Kp3(10) is followed by a GKK motif (Figure A3 in Appendix). Seven amino-acids up-stream the sequence of the Kp3(10), a stop codon appears (Figure A3 in Appendix) suggesting that coelacanth *Kiss3* gene could have a different intro-exon structure compared to what has been described so far or it can suggest that this gene is no longer expressed.

*A third *Kiss* gene in the elephant shark genome*. While two *Kiss* (*Kiss1* and *Kiss2*) were previously identified in the elephant shark genome (Lee et al., 2009), we were able to localize a new ORF of 315 bp containing a third sequence encoding a Kp(10) (Figure A4 in Appendix). Once translated, it leads to a peptidic sequence encompassing a putative Kp(10): YNLNSFGLKF [elephant shark Kp3(10)] (Figure 4). The C-terminal end of this peptide is followed by a GKR motif (Figure A4 in Appendix).

##### Kiss sequence alignment and comparisons

The alignment of 56 kisspeptin precursors revealed a high variability of their amino-acid sequences except for the sequences corresponding to Kp(10) and its few surrounding amino-acids which, in contrast, are highly conserved (data not shown). Such a pattern, which is representative of many other neuropeptide precursors, provides poor phylogenetic information within alignment matrix. This lack of information makes the use of phylogenetic analysis inappropriate to establish homology relationships within this kind of peptide precursor family.

##### Novel Kp(10)

Among the new Kiss genes predicted in the present study, four of them encode novel Kp(10) (Figure 4). The singularity of the elephant shark Kp3(10) is the presence of a lysine (K) instead of an arginine (R) at the ninth position. The coelacanth Kp1(10) provides the first case of a threonine (T) at the fifth position. The coelacanth Kp3(10) is the only one to present both a phenylalanine (F) at the first position and a serine (S) at the fifth position. The European eel Kp2(10) presents at its third position an arginine (R), which possesses different physical and chemical properties from amino-acids commonly present at this position. Up to now, only the musk shrew (*Suncus murinus*) Kp1(10) presented an arginine at the third position and it was demonstrated that its kisspeptin system was involved in the reproductive function as in other mammals (Inoue et al., 2011). Since the impacted positions by the amino-acid substitutions have not been characterized as highly critical for Kp(10) functional properties (Gutiérrez-Pascual et al., 2009; Curtis et al., 2010), those novel Kp(10) may have conserved their functionality. Since Kp(10) is considered as the smallest required sequence to specifically bind to the receptor (Kotani et al., 2001), it could be of interest to test all those peptides in future pharmacological studies in order to assess their structure/function relationships.

##### Syntenic analysis and classification of *Kiss* genes

In order to classify the different *Kiss* paralogs, we performed a syntenic analysis of the *Kiss* neighboring genes. We considered the following vertebrate representatives: mammals (human), birds (chicken), squamates (lizard), amphibians (*Xenopus*), basal sarcopterygian (coelacanth), non-teleost actinopterygians (spotted gar), and teleosts (zebrafish, stickleback, and European eel). Our syntenic analysis demonstrated that the *Kiss* genes are localized in three different genomic regions.

**Figure 5:**
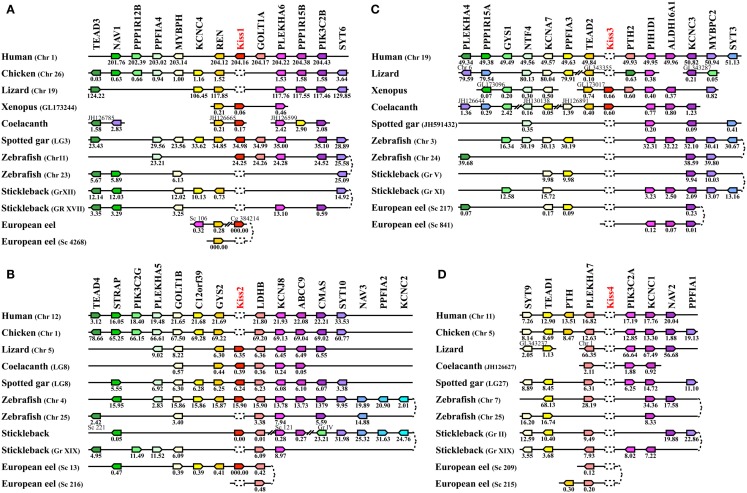
**Conserved genomic synteny of osteichthyan *Kiss***. Genomic synteny maps comparing the orthologs of *Kiss1* **(A)**, *Kiss2* **(B)**, *Kiss3* **(C)**, *Kiss4* **(D)**, and their neighboring genes. Analyses were performed on the genomes of human (*Homo sapiens*), platypus (*Ornithorhynchus anatinus*), lizard (*Anolis carolinensis*), chicken (*Gallus gallus*), *Xenopus* *(Xenopus tropicalis*), coelacanth (*Latimeria chalumnae*), spotted gar (*Lepisosteus oculatus*), zebrafish (*Danio rerio*), stickleback (*Gasterosteus aculeatus*), and European eel (*Anguilla anguilla*). This map was established using the PhyloView of Genomicus v67.01 web site, manual annotation of the European eel genome using CLC DNA Workbench 6 software and the gene annotation of the coelacanth and spotted gar genomic databases. *Kiss* genes are named according to our proposed nomenclature (*Kiss1* to *Kiss4*). The other genes are named after their human orthologs according to the Human Genome Naming Consortium (HGNC). Orthologs of each gene are shown in the same color. The direction of arrows indicates the gene orientation, with the position of the gene (in 10^−6^ base pairs) indicated below. The full gene names and detailed genomic locations are given in Table S1 in Supplementary Material. Chr, chromosome; LG, linkage group; Gr, group; Sc, scaffold; Cg, contig.

The human *Kiss1*, *Xenopus* *Kiss1a*, coelacanth *Kiss1*, spotted gar *Kiss1*, and zebrafish *Kiss1* are positioned in genomic regions containing common loci, including *TEAD3*, *NAV1*, *PPP1R12B*, *PPFIA4*, *MYBPH*, *KCNC4*, *REN*, *GOLT1A*, *PLEKHA6*, *PPP1R15B*, *PIK3C2B*, and *SYT6*, thus exhibiting well conserved synteny (Figure 5A). This supports the orthology of these *Kiss* genes, all considered as *Kiss1* genes. Syntenic analysis suggests that the stickleback, lizard, and chicken genomes do not contain any *Kiss1* gene, although the above-mentioned neighboring genes are present in the corresponding genomic regions (Figure 5A). The peptidic sequence of eel Kiss1 presents many similarities to the other Kiss1, but the eel *Kiss1* gene is located on too small scaffolds to contain any other gene, preventing from any syntenic analysis.

The lizard *Kiss2*, coelacanth *Kiss2*, spotted gar *Kiss2*, zebrafish *Kiss2*, stickleback *Kiss2*, and European eel *Kiss2* genes are positioned in genomic regions containing common loci including *STRAP*, *PLEKHA5*, *GOLT1B*, *C12orf39*, *GYS2*, *LDHB*, *KCNJ8*, *ABCC9*, *CMAS*, *SYT10*, *NAV3*, *PPFIA2*, and *KCNC2*, thus exhibiting well conserved synteny (Figure 5B). This supports the orthology of these *Kiss* genes, all considered as *Kiss2* genes. Syntenic analysis suggests that human and chicken genomes do not contain any *Kiss2* gene, although the above-mentioned neighboring genes are present in the corresponding genomic region (Figure 5B).

The coelacanth *Kiss3* and the *Xenopus* *Kiss1b* genes are positioned in genomic regions containing common loci, including *TEAD2*, *PIH1D1*, and *ALDH16A1*, thus exhibiting well conserved synteny (Figure 5C). This supports the orthology of these two *Kiss* genes, both considered here as *Kiss3* genes. Syntenic analysis suggests that human, lizard, spotted gar, and teleost genomes do not contain any *Kiss3* gene, although the above-mentioned neighboring genes are present in the corresponding genomic regions (Figure 5C). Syntenic analysis also suggests that the whole considered region is absent from the chicken genome.

#### Evolutionary history of *Kiss*

##### Origin of *Kiss* diversity via 1R and 2R

The syntenic analysis also allowed us to investigate the origin of the multiple *Kiss* genes found in vertebrates. The three conserved genomic regions, presenting *Kiss* genes, also comprise other paralogs from 11 gene families: *TEAD* (4 paralogs), *NAV* (3 paralogs), *PPFIA* (4 paralogs), *KCNC* (4 paralogs), *GOLT1* (2 paralogs), *PLEKHA* (4 paralogs), *PPP1R15* (2 paralogs), *PIK3C2* (3 paralogs), *SYT* (4 paralogs), *GYS* (2 paralogs), and *PTH* (2 paralogs) (Figures 5A–C). The members of those families are present among the three *Kiss* syntenic regions and they also delineate a fourth conserved region (Figure 5D), which does not present any *Kiss* gene in the osteichthyan representative species studied so far. The four conserved regions delineated by the 11 gene families can be considered as paralogous (tetra-paralogon).

**Figure 6:**
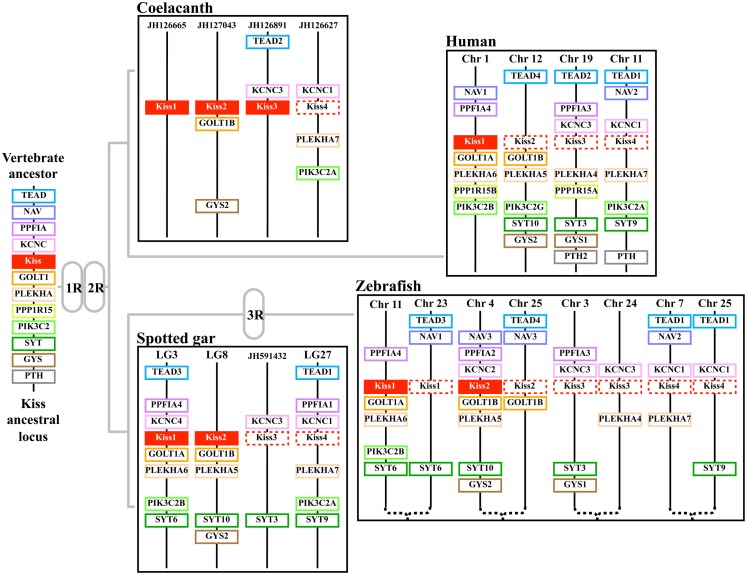
**Proposed origin of osteichthyan *Kiss* tetra-paralogons**. The paralogous genes of each of the 11 identified families delineate four paralogons in spotted gar, coelacanth, and human genomes and a duplicated tetra-paralogon in the zebrafish genome. This suggests a common origin of the three *Kiss* before the two whole genome duplication rounds (1R and 2R) which occurred in the early vertebrate history. This also suggests no impact of the teleost-specific 3R on *Kiss* number in current teleost species. Chr, chromosome; LG, linkage group.

**Figure A1:**
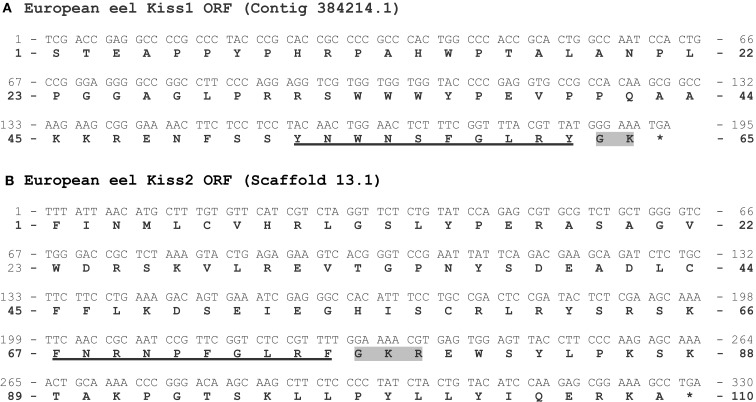
**Prediction of two *Kiss* ORFs from the European eel genome**. Nucleotide and deduced amino-acid sequences of the ORF encoding the European eel Kp1(10) **(A)** and Kp2(10) **(B)**. Nucleotides (top) are numbered from 5′ to 3′. The amino-acid residues (bottom) are numbered beginning with the first amino-acid residue encoded by the ORF. The asterisk (*) indicates the stop codon. The predicted Kp(10) are underlined. The amino-acids of the C-terminal α-amidation and cleavage site are shaded in gray.

**Figure A2:**
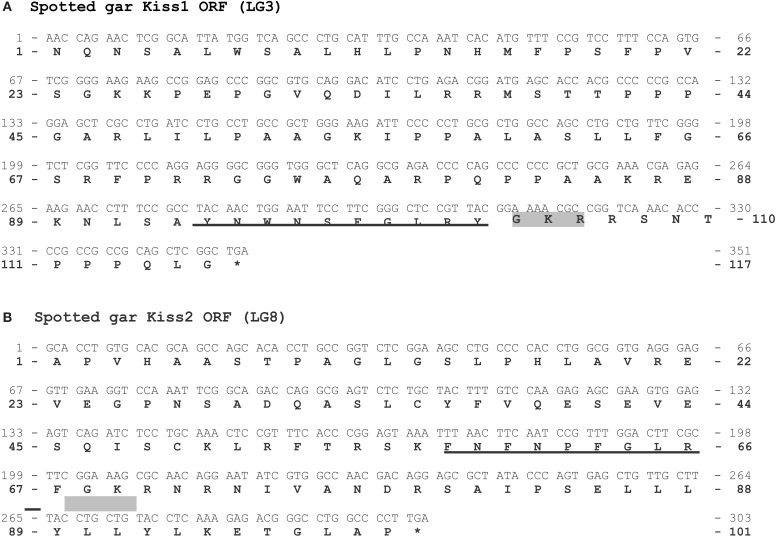
**Prediction of two *Kiss* ORFs from the spotted gar genome**. Nucleotide and deduced amino-acid sequences of the ORF encoding the spotted gar Kp1(10) **(A)** and Kp2(10) **(B)**. Nucleotides (top) are numbered from 5′ to 3′. The amino-acid residues (bottom) are numbered beginning with the first amino-acid residue encoded by the ORF. The asterisk (*) indicates the stop codon. The predicted Kp(10) are underlined. The amino-acids of the C-terminal α-amidation and cleavage site are shaded in gray.

**Figure A3:**
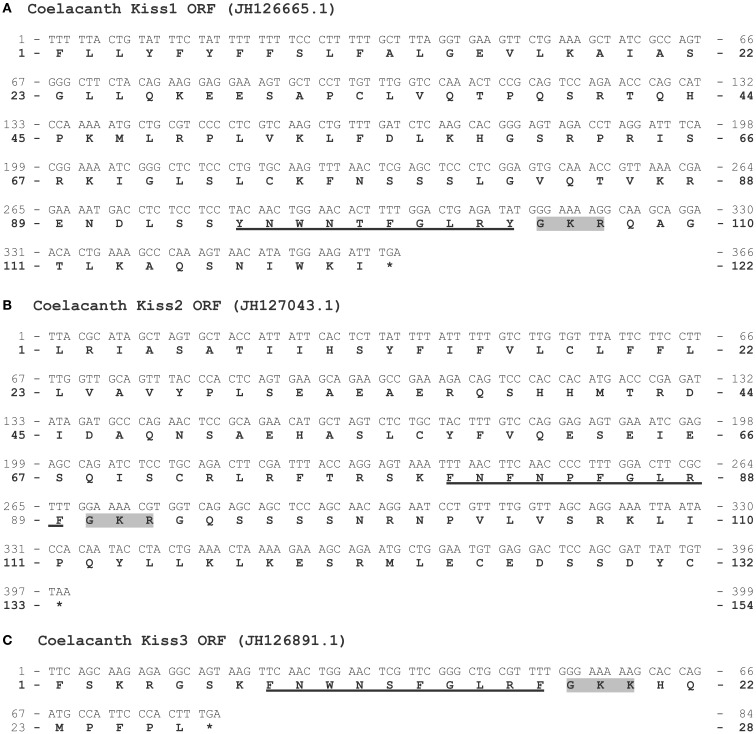
**Prediction of three *Kiss* ORFs from the coelacanth genome**. Nucleotide and deduced amino-acid sequences of the ORF encoding the coelacanth Kp1(10) **(A)**, Kp2(10) **(B)**, and Kp3(10) **(C)**. Nucleotides (top) are numbered from 5′ to 3′. The amino-acid residues (bottom) are numbered beginning with the first amino-acid residue encoded by the ORF. The asterisk (*) indicates the stop codon. The predicted Kp(10) are underlined. The amino-acids of the C-terminal α-amidation and cleavage site are shaded in gray.

**Figure A4:**
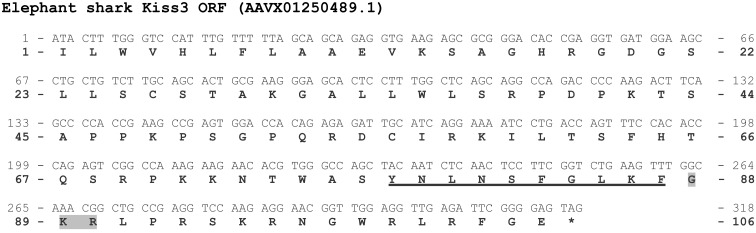
**Prediction of a third *Kiss* ORF from the elephant shark genome**. Nucleotide and deduced amino-acid sequences of the ORF encoding the elephant shark Kp3(10). Nucleotides (top) are numbered from 5′ to 3′. The amino-acid residues (bottom) are numbered beginning with the first amino-acid residue encoded by the ORF. The asterisk (*) indicates the stop codon. The predicted Kp(10) is underlined. The amino-acids of the C-terminal α-amidation and cleavage site are shaded in gray.

Considering the reconstruction of the ancestral vertebrate chromosomes, their linkage to the tetra-paralogons in the human genome (Nakatani et al., 2007) and our localization of the four *Kiss* syntenic regions in the human genome (on Chromosomes 1, 11, 12, and 19), we can hypothesize that the *Kiss* tetra-paralogons resulted from the duplications of one single genomic region localized on the proto-chromosome-D of the vertebrate ancestor. Therefore, we can infer that the current three *Kiss* genes may have resulted from a single ancestral gene duplicated through 1R and 2R that occurred in early steps of vertebrate evolution (Figure 6).

##### A subsequent history of *Kiss* losses

*Multiple loss events in sarcopterygians and actinopterygians*. The 1R and 2R events should have resulted in four different *Kiss* genes in vertebrates. Since the fourth *Kiss* gene (referred to as *Kiss4* in this study) has not been observed in any species studied so far, we can hypothesize an early loss of this gene after the 2R. As only a chondrichthyan, the elephant shark, and two sarcopterygians, the coelacanth and *Xenopus*, still present three *Kiss* genes, whereas all other species possess less than three *Kiss*, we can hypothesize multiple additional events of *Kiss* losses in vertebrates (Figure 3B).

Among the sarcopterygian lineage, in tetrapods, *Kiss3* would have been lost in amniotes. Further alternative losses may have occurred in this lineage, with only *Kiss1* remaining in eutherian mammals and only *Kiss2* in squamates (lizard). Finally, additional losses would have led to the complete absence of *Kiss* in birds (Figure 3B). Among the actinopterygian lineage, an early loss of *Kiss3* would have occurred since it is lacking in the actinopterygian species investigated so far (Figure 3B).

*No impact of the teleost-specific 3R on *Kiss* number in current species*. In the actinopterygian lineage, the teleost-specific 3R and the presence of two *Kiss* in a non-teleost actinopterygian, the spotted gar, implied the potential existence of at least four *Kiss* genes in the early teleost history. However, our study showed that so far the largest number of *Kiss* exhibited by current teleosts, including the eel, is two. Furthermore, each teleost *Kiss* is orthologous to a different tetrapod *Kiss,* indicating that no teleost-specific *Kiss* exists. Synteny analysis revealed that each of the four *Kiss* genomic regions present in the spotted gar is duplicated in teleosts in agreement with the 3R event but that duplicated *Kiss* genes are lacking (Figures 5 and 6). This suggests an early loss of duplicated *Kiss* genes suppressing the impact of the 3R on the number of *Kiss* in teleosts (Figure 3B). Additional deletion may have led to the presence of only *Kiss2* in gasterosteiforms (the stickleback; Figure 3B). *Kiss* evolutionary history was punctuated by numerous loss events through vertebrate radiation (Figure 3B).

#### Comparison of the evolutionary histories of *Kiss* and *Kissr* in vertebrates

These new data concerning *Kiss* and *Kissr* diversities enabled us to improve their respective classifications and evolutionary histories. A remaining challenge was to elucidate whether *Kiss* and *Kissr* families have experienced parallel histories during vertebrate radiation. The comparative study of the current status of both families in vertebrates allows a better understanding of the whole kisspeptin system.

##### Features in agreement with parallel histories

###### Origin of the *Kiss* and *Kissr* multiplicity via 1R and 2R

Our syntenic studies suggest that the vertebrate *Kiss* and *Kissr* families both resulted from the successive duplications of a single ancestral gene through the 1R and 2R (Figure 3). Thus, *Kiss* and *Kissr* experienced, in parallel, the two first genome duplication rounds resulting in four copies of each gene in the early steps of the vertebrate evolutionary history (Figure 3). While *Kissr* gene homologs were characterized in non-vertebrate species (*Strongylocentrotus purpuratus*, GenBank accession numbers: XP_793873.1 and XP_796286.1; *Saccoglossus kowalevskii*, GenBank accession numbers: NP_001161573.1 and NP_001161574.1), tracing back the presence of an ancestral *Kissr* in early deuterostomes, *Kiss* genes have not yet been discovered in non-vertebrate species.

###### Subsequent history of gene losses

Both *Kiss* and *Kissr* families were composed of four genes in the early steps of the vertebrate history. However, most of the current vertebrate species investigated so far present less than four copies of *Kiss* and *Kissr* genes. The current numbers of both *Kiss* and *Kissr* genes suggest that both families underwent numerous independent loss events across vertebrate history (Figure 3).

###### No impact of the teleost-specific 3R

The teleost lineage, which has experienced a third whole genome duplication round (3R), could have been expected to possess up to eight *Kiss* and *Kissr* genes. However, the analyses of the *Kiss* and *Kissr* within teleost genomes revealed a maximum of three *Kissr* and two *Kiss* genes and did not reveal any 3R-specific copies of *Kiss* or *Kissr* genes. This suggests that the teleost-specific 3R did not impact the current number of *Kiss* or *Kissr* genes, reflecting massive losses of the 3R-copies of both *Kiss* and *Kissr* genes in early teleosts (Figure 3).

##### Features in opposition to parallel histories: independent loss events

##### Un-matching number of *Kiss* and *Kissr* in some species

In the current gnathostomes, we observed a maximum of four *Kissr* but only three *Kiss* paralogs. This difference suggests that this lineage inherited the four *Kissr* copies resulting from the 1R and 2R, whereas the fourth *Kiss* may have been lost before or at an early stage of the gnathostome emergence (Figure 3). This situation was observed in an early sarcopterygian, the coelacanth, while an even larger difference in *Kissr* (four) and *Kiss* (two) numbers was found in the spotted gar, reflecting an additional independent loss of *Kiss* in the actinopterygian lineage. An un-matching number of *Kissr* (three) and *Kiss* (two) was also observed in an early teleost, the European eel, while additional losses may have led to equal numbers of *Kissr* and *Kiss* in more recent teleosts (two or one according to the species). These variations in *Kissr/Kiss* numbers reflect different timing of *Kiss* and *Kissr* loss events. Those hypotheses suggest that *Kiss* losses occurred independently among the different gnathostome lineages and also independently from the *Kissr* losses.

##### Various *Kiss*/*Kissr* combinations across vertebrates

The hypothesis of independent *Kiss* and *Kissr* evolutionary histories is also strengthened by the comparison of the gene sets present in species with even matching numbers of *Kiss* and *Kissr*. For example, lizard, and stickleback both present the *Kiss2* gene, whereas they possess different *Kissr*, i.e., *Kissr-4* in the lizard and *Kissr-2* in the stickleback (Figure 3). The same observation can be done comparing the kisspeptin systems of platypus and zebrafish, which both present the *Kiss1* and *Kiss2* genes, whereas their sets of receptors are completely different, i.e., *Kissr-1* and *Kissr-4* in platypus versus *Kissr-2* and *Kissr-3* in zebrafish (Figure 3). These observations strongly suggest a large variety of *Kiss* and *Kissr* combinations, resulting from independent loss events. These data shed new lights on the evolution of the kisspeptin system in vertebrates and challenge the former hypothesis of a conservation of *Kiss/Kissr* couples across vertebrate evolution. This diversity among vertebrates opens new research avenues for comparative physiology and endocrinology of kisspeptin system.

##### What could have favored the independent evolutions of *Kiss* and *Kissr*?

A few *in vitro* studies using recombinant receptors have showed cross-reactivities between various kisspeptins and kisspeptin receptors (Biran et al., 2008;Lee et al., 2009; Li et al., 2009). For instance, Lee et al. (2009) tested the specificity of recombinant human GPR54 (Kissr-1 according to our nomenclature), zebrafish GPR54-1, and -2 (Kissr-3 and Kissr-2 according to our nomenclature), and *Xenopus* GPR54-1a, -1b, and -2 (Kissr-1, Kissr-4, and Kissr-2 according to our nomenclature) toward various kisspeptins. They showed that human, zebrafish, and *Xenopus* kisspeptins were able to activate all the receptors with differential intra and inter-specific ligand selectivity. Such cross-reactivity could have promoted the independence of the *Kiss* and *Kissr* evolutionary histories. This could explain the situation of species presenting un-matching numbers of *Kiss* and *Kissr* genes, as well as the high variability of *Kiss/Kissr* gene combinations across vertebrate species. Another study, using goldfish recombinant GPR54a and GPR54b (Kissr-3 and Kissr-2 according to our nomenclature), revealed that the ligand potency strikingly differed depending on the responsive element used in the reporter gene construction (Li et al., 2009). These data showed the difficulty to define specific Kiss/Kissr couples based only on pharmacological properties.

In the case of the presence of multiple *Kiss/Kissr* genes in a given species, anatomical relationships between projections of *Kiss* neurons and target cells expressing *Kissr* may provide further cues for determining Kiss/Kissr functional couples. Thus, in zebrafish which possess two *Kiss* genes and two *Kissr* genes, *in situ* hybridization and immunocytochemical studies localized the *Kiss1* neurons in different nuclei from *Kiss2* neurons (Servili et al., 2011). Moreover, *Kiss1* neurons are projecting to *Kiss1r* (*Kissr-3* according to our nomenclature) expressing cells, while *Kiss2* neurons are projecting to *Kiss2r* (*Kissr-2* according to our nomenclature) expressing cells (Servili et al., 2011). This reveals anatomically separated kisspeptin systems and distinct specific Kiss/Kissr functional couples in zebrafish (Servili et al., 2011). In striped bass, another teleost possessing two *Kiss* genes and two *Kissr* genes, *in situ* hybridization and laser capture microscopy coupled to quantitative PCR showed, in contrast, that *Kiss1* and *Kiss2* were co-expressed in neurons of the hypothalamus, indicating promiscuous Kiss synthesis sites (Zmora et al., 2012). However, the two *Kissr* of the striped bass were expressed in different brain cells, indicating that the kisspeptin systems are not fully redundant (Zmora et al., 2012). All these data underline the importance of investigating the gene diversity, the anatomical organization and the functional properties of the kisspeptin system in various species, regarding the potential high variability of this system among vertebrates.

## Conclusion

Kisspeptin system is known to play a role in many physiological processes such as antimetastasis, energy metabolism homeostasis, pregnancy, and puberty onset. Even though this system has been widely studied in the last few years, its diversity and evolutionary history remained unclear. Thanks to the newly published genomes of osteichthyans of key phylogenetical positions, we were able to provide new data on the diversity of *Kiss* and *Kissr* genes, to clarify the classification of these genes and to bring new insights on the evolutionary history of these gene families. Four *Kissr* and four *Kiss* genes may have arisen via the 1R and 2R in early vertebrates. This would have been followed by multiple independent *Kiss* and *Kissr* gene loss events in the sarcopterygian and actinopterygian lineages. In particular, due to massive *Kiss* and *Kissr* gene losses, no impact of the teleost-specific 3R can be recorded on the number of *Kissr* or *Kiss* paralogs in current teleost species. The comparison of both *Kiss* and *Kissr* gene status, in the current vertebrates, supports both parallel and independent evolutionary histories of the *Kiss* and *Kissr* families across vertebrate radiation. It also underlines a large diversity of *Kiss*/*Kissr* possible combinations that needs to be taken into account in future comparative studies.

## Conflict of Interest Statement

The authors declare that the research was conducted in the absence of any commercial or financial relationships that could be construed as a potential conflict of interest.

## Supplementary Material

The Supplementary Material for this article can be found online at http://www.frontiersin.org/Neuroendocrine_Science/10.3389/fendo.2012.00173/abstract

Supplementary Table S1 **Names, references, and locations of the genes used in the *Kiss* synteny analysis**.

## Footnotes

^1^http://www.ensembl.org/Gallus_gallus/Info/Index/

^2^http://www.ensembl.org/Latimeria_chalumnae/Info/Index/

^3^http://esharkgenome.imcb.a-star.edu.sg/resources.html

^4^http://www.zfgenomics.org/sub/eel

^5^http://www.ensembl.org/Homo_sapiens/Info/Index/

^6^http://www.ensembl.org/Anolis_carolinensis/Info/Index/

^7^http://www.ensembl.org/Ornithorhynchus_anatinus/Info/Index/

^8^http://www.ensembl.org/Petromyzon_marinus/Info/Index/

^9^http://pre.ensembl.org/Lepisosteus_oculatus/Info/Index

^10^http://www.ensembl.org/Gasterosteus_aculeatus/Info/Index/

^11^http://www.ensembl.org/Xenopus_tropicalis/Info/Index/

^12^http://www.ensembl.org/Danio_rerio/Info/Index/

^13^http://www.ensembl.org/index.html

^14^http://www.dyogen.ens.fr/genomicus-67.01/cgi-bin/search.pl
